# Fusion to Snowdrop Lectin Magnifies the Oral Activity of Insecticidal ω-Hexatoxin-Hv1a Peptide by Enabling Its Delivery to the Central Nervous System

**DOI:** 10.1371/journal.pone.0039389

**Published:** 2012-06-22

**Authors:** Elaine C. Fitches, Prashant Pyati, Glenn F. King, John A. Gatehouse

**Affiliations:** 1 Department for Environment, Food and Rural Affairs, Food and Environmental Research Agency, Sand Hutton, United Kingdom; 2 School of Biological and Biomedical Sciences, University of Durham, Durham, United Kingdom; 3 Division of Chemical and Structural Biology, Institute for Molecular Bioscience, University of Queensland, Brisbane, Australia; University of Kentucky, United States of America

## Abstract

**Background:**

The spider-venom peptide ω-hexatoxin-Hv1a (Hv1a) targets insect voltage-gated calcium channels, acting directly at sites within the central nervous system. It is potently insecticidal when injected into a wide variety of insect pests, but it has limited oral toxicity. We examined the ability of snowdrop lectin (GNA), which is capable of traversing the insect gut epithelium, to act as a “carrier” in order to enhance the oral activity of Hv1a.

**Methodology/Principal Findings:**

A synthetic Hv1a/GNA fusion protein was produced by recombinant expression in the yeast *Pichia pastoris*. When injected into *Mamestra brassicae* larvae, the insecticidal activity of the Hv1a/GNA fusion protein was similar to that of recombinant Hv1a. However, when proteins were delivered orally via droplet feeding assays, Hv1a/GNA, but not Hv1a alone, caused a significant reduction in growth and survival of fifth stadium *Mamestra brassicae* (cabbage moth) larvae. Feeding second stadium larvae on leaf discs coated with Hv1a/GNA (0.1–0.2% w/v) caused ≥80% larval mortality within 10 days, whereas leaf discs coated with GNA (0.2% w/v) showed no acute effects. Intact Hv1a/GNA fusion protein was delivered to insect haemolymph following ingestion, as shown by Western blotting. Immunoblotting of nerve chords dissected from larvae following injection of GNA or Hv1a/GNA showed high levels of bound proteins. When insects were injected with, or fed on, fluorescently labelled GNA or HV1a/GNA, fluorescence was detected specifically associated with the central nerve chord.

**Conclusions/Significance:**

In addition to mediating transport of Hv1a across the gut epithelium in lepidopteran larvae, GNA is also capable of delivering Hv1a to sites of action within the insect central nervous system. We propose that fusion to GNA provides a general mechanism for dramatically enhancing the oral activity of insecticidal peptides and proteins.

## Introduction

Arthropod venoms contain a rich diversity of compounds including a significant number of neurotoxic disulphide-rich peptides. Most spiders prey exclusively upon insects and other arthropods and thus it is not surprising that many spider-venom peptides have been shown to modulate the activity of arthropod ion channels. One such example is ω-hexatoxin-Hv1a (formerly ω-atracotoxin-Hv1a; hereafter referred to as Hv1a), the best-studied member of a family of 36–37 residue insecticidal neurotoxins isolated from the venom of the Australian funnel web spider *Hadronyche versuta.* Hv1a specifically inhibits insect but not mammalian voltage-gated calcium channels [1], [2], [3]. Structurally, Hv1a comprises a disordered N-terminus (residues 1–3), a disulfide-rich globular core (residues 4–21), and a highly conserved C-terminal β hairpin (residues 22–37) that protrudes from the disulphide-rich core and contains the key residues for insecticidal activity [4], [2]. The three disulphide bonds form an inhibitor cystine knot motif that provides many spider-venom peptides with extreme chemical and thermal stability, as well as resistance to proteases [5], [6].

Hv1a is highly toxic by injection towards many different insect pests including species from the Orders Lepidoptera, Coleoptera, Dipteran and Dictyoptera [7]–[10]. Its potency and phyletic specificity makes Hv1a an ideal candidate for development of novel bioinsecticides. However, whilst toxic by injection, Hv1a and many other insecticidal venom peptides are typically ineffective, or at least much less potent, when delivered orally and this is thought to be due to ineffective delivery of the toxins to their sites of action in the central (CNS) or peripheral nervous system (PNS). This lack of oral activity clearly limits their potential application as bioinsecticides. In order to access the nervous system after oral delivery, peptide toxins must be resistant to proteolytic degradation in the insect gut, and they must be able to cross the insect gut epithelium. The latter factor is thought to be the major limitation in oral toxicity of protein and peptide toxins.

The mannose-specific lectin GNA (*Galanthus nivalis* agglutinin; snowdrop lectin) is resistant to proteolytic activity in the insect gut. Moreover, following ingestion, GNA binds to gut epithelial glycoproteins and is transported into the haemolymph [11]. This property of GNA can be used to transport peptides across the insect gut [12]. Previous results have shown that the oral insecticidal activity of peptides derived from the venom of spiders and scorpions can be dramatically enhanced by fusing them to GNA [13]–[16], presumably because GNA mediates their delivery to the hemolymph where the toxins can subsequently reach their sites of action in the CNS or PNS.

The present paper reports on the insecticidal activity of a fusion protein comprised of Hv1a linked to the N-terminus of GNA. We show that the Hv1a/GNA fusion protein, expressed in yeast and purified from culture supernatant, is biologically active by injection, indicating that fusion to GNA does not compromise the insecticidal activity of Hv1a. Whereas Hv1a alone was not orally active against the cabbage moth *Mamestra brassicae*, the Hv1a/GNA fusion protein had significant oral activity against this lepidopteran crop pest. Moreover, for the first time, we present direct evidence for binding of orally delivered GNA to the CNS of lepidopteran larvae. This suggests that in addition to providing a mechanism for delivery of peptide toxins across the insect gut, GNA may further facilitate toxin activity by delivering covalently attached toxins to the CNS of insects. Thus, fusion to GNA provides a general mechanism for dramatically enhancing the oral activity of insecticidal peptide neurotoxins.

## Results

### Synthetic Gene and Fusion Protein Construct Assembly

**Figure 1:**
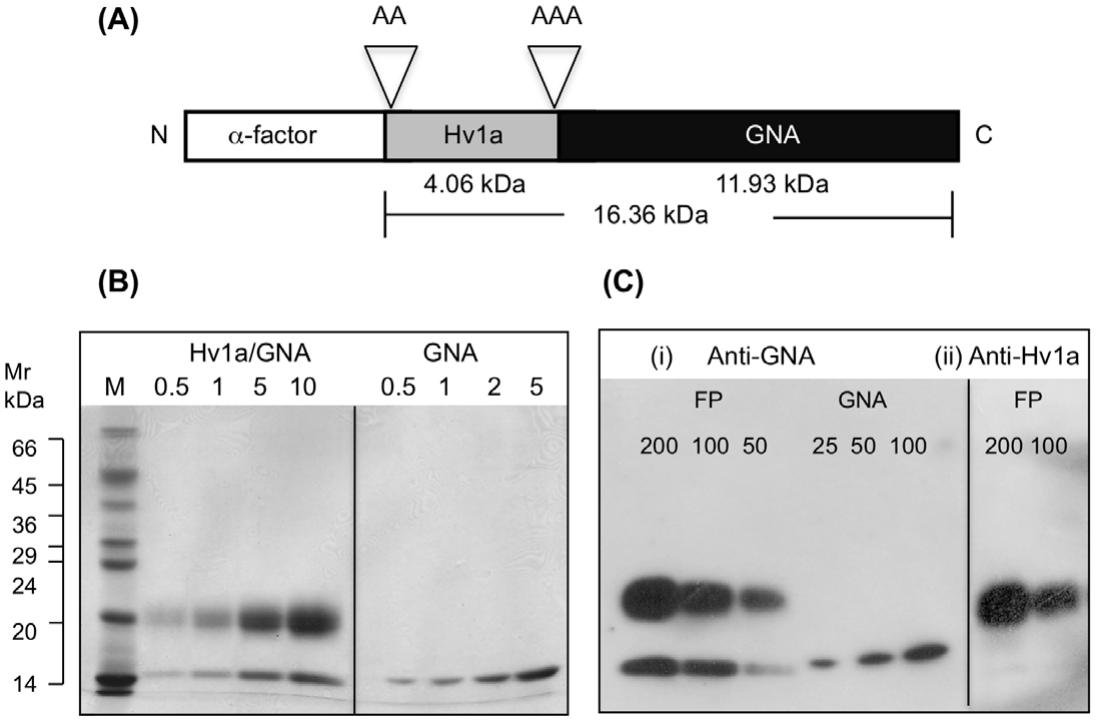
Protein production and purification. (A) Schematic of construct encoding Hv1a/GNA showing predicted molecular masses of Hv1a and GNA as well as the total mass of the Hv1a/GNA fusion protein including the tri-alanine linker region and the additional two alanine residues at the N-terminus. (B) Coomassie blue stained SDS-PAGE gel (17.5% acrylamide) of recombinant Hv1a/GNA and GNA following purification by hydrophobic interaction and gel filtration chromatography. The approximate loading of protein (µg) is indicated above each lane, while the lane marked “M” contains molecular weight standards (Sigma SDS-7). (C) Composite of Western blots of recombinant proteins using (i) anti-GNA and (ii) anti-Hv1a antibodies. The approximate protein loading (ng) is denoted above each lane. FP denotes Hv1a/GNA fusion protein.

A synthetic gene encoding the mature Hv1a amino acid sequence was assembled using a series of overlapping oligonucleotides, with codon usage optimised for expression in yeast (Table 1). Following assembly, the coding sequence was amplified by PCR and ligated into a yeast expression vector (derived from pGAPZαB) that contained a sequence coding for the mature GNA polypeptide (amino acid residues 1–105). The 37-residue Hv1a peptide was fused to the N-terminus of GNA via a tri-alanine linker sequence as depicted in Fig. 1A. The Hv1a/GNA construct was cloned such that the N-terminal yeast α-factor prepro-sequence would direct the expressed protein to the yeast secretory pathway. The final Hva1/GNA fusion protein is predicted to contain an additional two alanine residues at the N-terminus (after removal of the prepro- sequence) and terminate at residue 105 of the mature GNA protein, giving a predicted molecular mass of 16.36 kDa. The Hv1a/GNA-pGAPZαB construct was cloned into *E. coli* and the coding sequence was verified by DNA sequencing.

**Table 1:** Oligonucleotide sequences used for assembly and amplification of a synthetic gene encoding for the mature Hv1a toxin.

Coding strand
Oligo 1:
5′-GCATCTCCAACTTGTATTCCATCTGGTCAACCATGTCCATATAATGAAAATTGTTGT
Oligo 2:
5′-TCTCAATCTTGTACTTTTAAAGAAAATGAAAATGGTAATACTGTTAAAAGATGTGATGC
Complementary strand
Oligo 3:
ACGT CGTAGAGGTTGAACATAAGGTAGACCAGTTGGTACA
Oligo 4:
GGTATATTACTTTTAACAACAAGAGTTAGAACATGAAAATTT
Oligo 5:
CTTTTACTTTTACCATTATGACAATTTTCTACACTACGCCGG
Primers for amplification of full-length sequence
Forward primer:
5′ TAACTGCAGCATCTCCAACTTGTATTCC
Reverse primer:
5′ TTAGCGGCCGCATCACATCTTTTAACAG

Underlined bases depict restriction sites (*Pst*I and *Not* I) used for ligation of the full-length fragment into the yeast expression vector pGAPZαB.

### Expression and Purification of Recombinant Hv1a/GNA Fusion Protein

DNA from a verified Hv1a/GNA-pGAPZαB clone was linearised, transformed into the protease-deficient *P. pastoris* strain SMD1168H, and selected on antibiotic containing plates. Ten clones were analysed for expression of recombinant protein by Western blot (using anti-GNA antibodies) of supernatants derived from small-scale cultures (results not shown). This allowed selection of the best expressing clone for fusion protein production by bench-top fermentation.

For fusion protein production, *P. pastoris* cells were grown in a BioFlo 110 laboratory fermenter. Recombinant GNA was expressed and purified as previously described [14]. The Hv1a/GNA fusion protein was purified from clarified culture supernatant by hydrophobic interaction chromatography followed by a second gel-filtration step to remove high molecular weight contaminating yeast proteins. Two major proteins of ∼20 kDa and ∼14.5 kDa were recovered following fermentation and purification of recombinant Hv1a/GNA (Fig. 1B). The 20-kDa protein migrates at a higher than expected molecular weight than the 16.36 kDa predicted for intact fusion protein. However, Western blot analysis (Fig. 1C) using anti-GNA and anti-Hv1a antibodies confirmed that the higher molecular weight protein represents intact fusion protein as it is immunoreactive with both anti-GNA and anti-Hv1a antibodies. The lower molecular weight band, which does not show positive immunoreactivity with anti-Hv1a antibodies, represents GNA from which the Hv1a peptide has been cleaved. Analysis of samples taken during fermentation confirmed that cleavage of the fusion protein occurs during expression and not during purification (results not shown). Intact Hv1a/GNA fusion protein was expressed at levels of ∼50 mg/l of culture supernatant. The ratio of intact fusion protein to cleaved GNA was consistently 2∶1 as judged by SDS-PAGE gels and Western blots.

### Injection Toxicity of Hv1a/GNA and Hv1a

The biological activity of Hv1a/GNA was verified by injection of 5–20 µg of purified fusion protein into fifth stadium *M. brassicae* larvae (40–70 mg). Injections of comparable molar amounts of recombinant Hv1a (2.3–9.2 µg) were also conducted. Larval mortality occurred over a period of 4 days (Table 2) but was observed predominantly within the first 48 h following injection.

**Table 2:** Mortality recorded for fifth stadium *M. brassicae* larvae 72 h after injection of different concentrations of recombinant Hv1a and Hv1a/GNA.

Treatment	Dose (µg/g insect) (Hv1a equivalents)	Mortality (%)	Sample No.
Control	–	0	20
Hv1a	184	90*	10
	92	80*	10
	46	20	10
Hv1a/GNA	100	90*	20
	50	45*	20
	25	0	20

Doses of injected Hv1a/GNA are expressed as Hv1a equivalents to allow a direct comparison with the Hv1a treatment and are based on a mean larval weight at injection of 50 mg. Asterisks denotes significant difference in survival between control and toxin treatment (P<0.0001).

Larvae injected with higher doses of fusion protein (10 µg and above) or toxin alone (4.6 µg and above) displayed symptoms of paralysis, and survival was significantly reduced as compared to the control treatment (Kaplan–Meier survival curves; Mantel–Cox log-rank tests; P<0.001). Levels of mortality were comparable between fusion protein injected and toxin injected treatments (e.g., 80% mortality for larvae injected with 92 µg toxin/g insect compared to 90% mortality for larvae injected with 100 µg toxin as a component of fusion protein/g insect).

### Oral Toxicity of Hv1a/GNA and Hv1a

**Figure 2:**
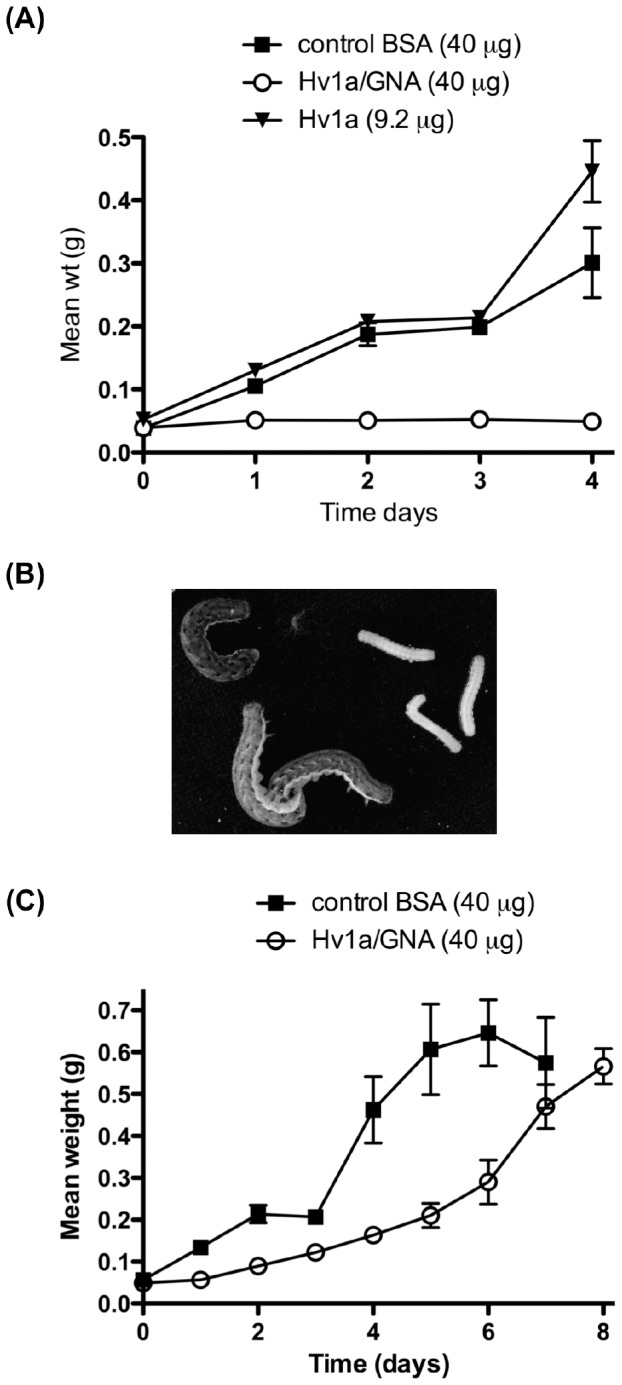
Droplet feeding assays. (A) Mean weight of fifth stadium *M. brassicae* larvae fed daily sucrose droplets containing either 9.2 µg Hv1a, 40 µg Hv1a/GNA, or 40 µg bovine serum albumin (BSA, control). Significant differences between Hv1a/GNA and control or Hv1a treatments were observed at days 1–4 (ANOVA Tukey post hoc; day 1, P = 0.0003; days 2–4, P<0.0001). (B) Image depicts larvae assayed in (A); control larvae on the left (BSA- and Hv1a-fed larvae) and Hv1a/GNA-fed larvae on the right. (C) Mean weight of fifth stadium *M. brassicae* larvae fed a single sucrose droplet containing either 40 µg Hv1a/GNA or control 40 µg BSA. Differences in mean weights between control and fusion protein treatments were significant from day 1 to day 6 of the assay (*t*-test; P<0.05).

Several experiments were performed to assess whether fusion to GNA was able to improve the oral toxicity of Hv1a. First, fifth stadium *M. brassicae* larvae were fed daily for four days on droplets containing 40 µg of purified fusion protein or 9.6 µg Hv1a (Fig. 2A). Ingestion of daily droplets of fusion protein was found to result in a complete cessation of larval feeding evidenced by the significantly reduced mean weight recorded for this treatment as compared to the control group. After four days, 40% of the treated larvae were dead and the remaining insects did not survive to pupation. In striking contrast, no reduction in larval growth as compared to the control BSA treatment was observed for larvae fed on droplets containing Hv1a, indicating that the oral toxicity of Hv1a is dramatically enhanced by fusion to GNA.

In a second assay, fourth stadium larvae were fed on a *single* droplet containing 40 µg of Hv1a/GNA (Fig. 2C) and this was shown to cause a reduction in larval growth as compared to control-fed larvae over a period of approximately six days. By day 7, control larvae had attained their maximum weight after which a reduction in weight was observed as insects enter the pre-pupal phase (day 6–7). By contrast, larvae that had ingested a single Hv1a/GNA-containing droplet exhibited a reduced growth rate reaching maximal weight at day 8–9, after which larvae pupated.

**Figure 3:**
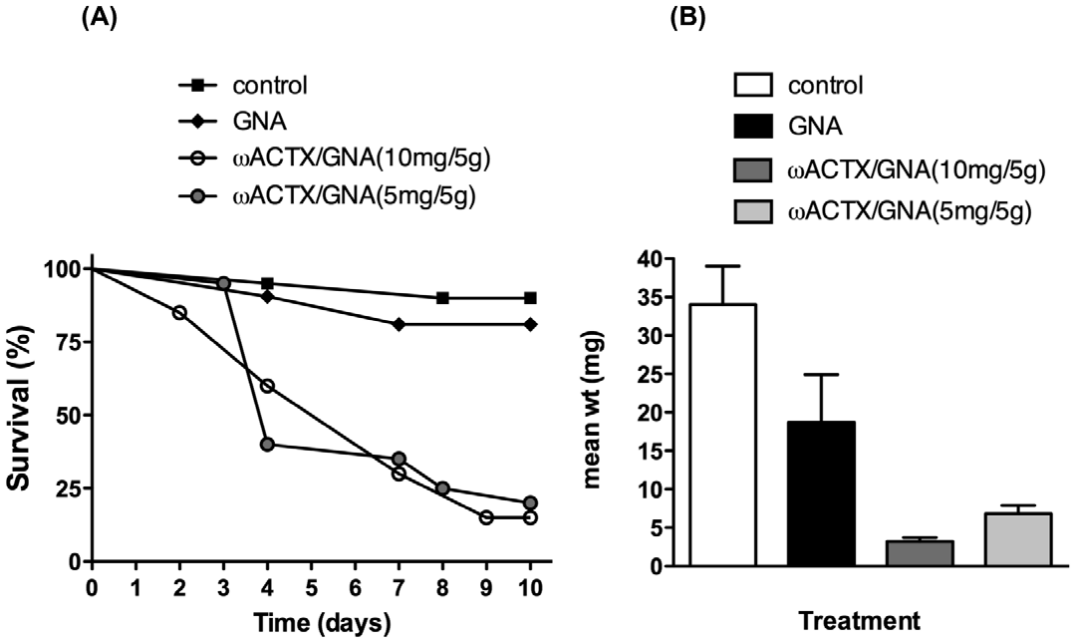
Leaf disc assays. (A) Survival of *M. brassicae* larvae fed from second stadia on cabbage discs coated with purified recombinant GNA (0.2% w/w) or Hv1a/GNA (0.2% w/w and 0.1% w/w) or on control PBS-coated discs (*n* = 20 per treatment). (B) Mean weight of larvae recorded at day 10.

The oral toxicity of the Hv1a/GNA fusion protein was further investigated by feeding 2nd instar *M. brassicae* larvae on cabbage discs coated with purified recombinant proteins, an assay that might be more representative of situations in which Hv1a is employed on crops as a foliar bioinsecticide. The survival of larvae was significantly reduced when insects were fed on Hv1a/GNA-coated discs (Fig. 3) such that 15% and 20% of larvae remained after 10 days of exposure to discs coated with Hv1a/GNA at concentrations of 0.2% w/w and 0.1% w/w, respectively. In contrast, 80% survival was recorded for larvae reared for 10 days on discs coated with 0.2% w/w GNA, which was not significantly different to the 90% survival recorded for the control (no added protein) treatment. Fusion protein treatment survival curves were significantly different to both the GNA and control treatments (Kaplan-Meier; Mantel–Cox log-rank tests; P<0.001). Exposure to Hv1a/GNA-coated discs also retarded larval growth in surviving larvae. The reduction in growth was dose-dependent, so that by day 7 the average weight of surviving larvae fed on 0.2% or 0.1% w/w Hv1a/GNA was reduced by 90% and 76%, respectively, compared to the control treatment. GNA was also shown to reduce larval growth, so that by day 7 the average weight of larvae fed 0.2% w/w GNA was reduced by 45% compared to the control treatment.

### Delivery of Ingested Hv1a/GNA to the Circulatory System and Binding of Injected Hv1a/GNA and GNA to the Central Nerve Chord

**Figure 4:**
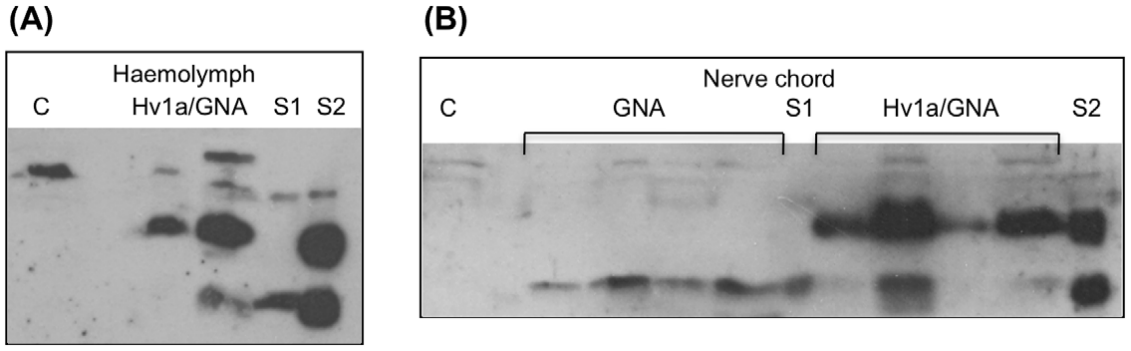
Analysis of haemolymph and nerve chords. Immunoblot analysis using anti-GNA antibodies of (A) haemolymph samples extracted from *M. brassicae* larvae 48 h after feeding on diet containing Hv1a/GNA (2 mg/5 g diet). “C” denotes control haemolymph (larvae fed on diet with no added protein). Lanes 1 and 2 are replicates of pooled samples (3 larvae per sample); 15 µl of haemolymph was loaded in all cases. (B) Nerve chord samples dissected from sixth stadium larvae that had been injected with 25 µg GNA (lanes 1–4) or Hv1a/GNA (lanes 5–8). Pooled samples were extracted 3 h (lanes 1, 2, 5, and 6) or 5 h (lanes 3, 4, 7, and 8) post injection. Pooled samples (4 nerve chords per sample) were extracted directly in 40 µl SDS-sample buffer and 20 µl was loaded per lane. “C” denotes control nerve chord sample. In panels (A) and (B), S1 and S2 are 50 ng standards of GNA and Hv1a/GNA respectively.

We have previously shown that GNA is capable of transporting covalently attached peptides across the insect gut into the hemolymph [11], [13]. To determine if the toxic effects observed in oral bioassays were attributable to GNA-mediated delivery of Hv1a to the circulatory system of *M. brassicae* larvae, haemolymph was extracted from insects fed on diets containing Hv1a/GNA and analysed for the presence of fusion protein by Western blotting using anti-GNA antibodies. A representative blot, depicted in Fig. 4A, confirms immunoreactivity of a major band corresponding to the molecular weight of intact fusion protein in samples from larvae fed Hv1a/GNA, but not control insects. As shown previously in Fig. 1C, fusion protein samples contain two GNA-immunoreactive bands corresponding to intact fusion protein and GNA from which the Hv1a peptide has been cleaved. Thus, the presence of a second smaller immunoreactive band in haemolymph samples from fusion protein fed larvae suggests uptake of both intact Hv1a/GNA and cleaved GNA, or cleavage of intact fusion protein after absorption in the insect gut. Cross-reactivity and poor sensitivity of the anti-Hv1a antibodies did not allow the detection of fusion protein or toxin when these antibodies were used to probe Western blots of larval haemolymph.

The above results indicate that the major reason for the improved oral activity of Hv1a when it is fused to GNA is the ability of this lectin to mediate delivery of Hv1a to the insect hemolymph. However, we also wondered whether GNA might also be able to enhance delivery of Hv1a to its sites of action in the insect nervous system. To investigate if GNA is able to bind to the nerve tract of lepidopteran larvae, intact nerve chords were dissected from insects injected with either GNA or Hv1a/GNA and analysed by Western blotting using anti-GNA antibodies. Nerve chords and haemolymph samples, pooled from 3–6 insects, were typically extracted 3–12 h following the injection of 10–20 µg of GNA or Hv1a/GNA. Fig. 4B shows positive immunoreactivity of bands corresponding in size to GNA and intact Hv1a/GNA fusion protein in both nerve chord and haemolymph samples taken from injected insects, which suggests that GNA is able to bind to the nerve tract of lepidopteran larvae. Bands corresponding to GNA or Hv1a/GNA fusion protein were not observed in nerve tissue extracted from insects fed on GNA or Hv1a/GNA (at 2.5 mg/5 g wet wt. diet), presumably due to the levels of bound protein being below the limits of detection of the anti-GNA antibodies.

**Figure 5:**
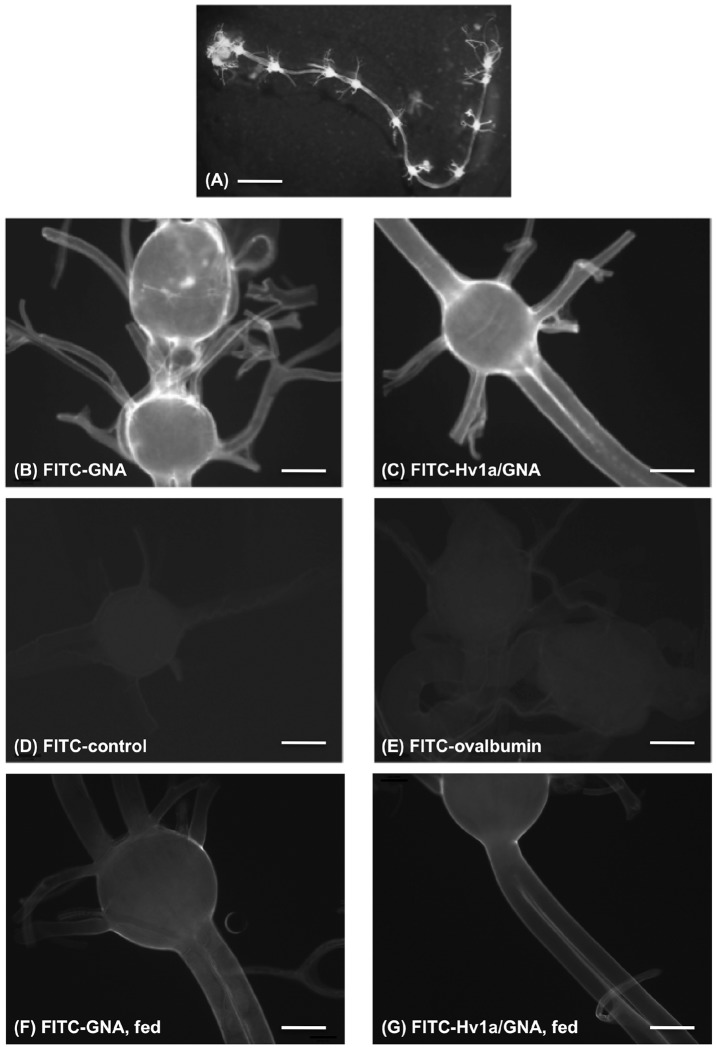
Binding of GNA to nerve chords. (A) Intact nerve chord dissected from sixth stadium *M. brassicae* larvae. (B–F) Composite of partial images of nerve tracts dissected from larvae injected with, or fed on, FITC-labelled proteins. Images were visualised with a fluorescent microscope under FITC filter and captured in OpenLab. B: FITC-GNA; C: FITC-Hv1a/GNA; D: control FITC; E: FITC-ovalbumin; F: FITC-GNA; G: FITC-Hv1a/GNA. Scale bar = 2 mm in (A) and 200 µM in (B–E).

Further evidence of the ability of GNA to bind to the central nerve chord was sought by visualisation of nerve chords dissected from insects that had been injected with, or fed on, fluorescently-labelled GNA or Hv1a/GNA. Control treatments were FITC-labelled ovalbumin or FITC alone. The visualisation of nerve chords dissected following injection was carried out on four separate occasions where typically 2–3 nerve chords per treatment were analysed and comparable results obtained. A composite showing different regions of *M. brassicae* nerve chords from different treatments is presented in Fig. 5. Low background fluorescence was observed in control FITC alone and FITC-labelled ovalbumin nerve chords. By contrast, fluorescence was observed along the entire length of the nerve tracts, including the terminal brain ganglion, of insects injected with FITC-labelled GNA or Hv1a/GNA. Fluorescence appeared to be predominantly localised to the nerve chord sheath. Reduced fluorescence was observed in instances where FITC-labelled GNA had been pre-incubated in the presence of mannose, suggesting that localisation to the nerve chord was mediated by binding of GNA to mannose-containing polypeptides in the nerve chord epithelium. However, binding was not completely inhibited under the conditions tested (results not shown). Similar results were obtained in experiments where larvae had been fed on diets containing FITC-labelled proteins although the levels of fluorescence were lower than those visualised from injected larvae (Fig. 5). This was attributed to lower levels of GNA and Hv1a/GNA being delivered to the circulatory system following ingestion as compared to the levels present in injected insects.

## Discussion

### Hva1 Retains Insecticidal Activity when Fused to GNA

Hv1a is the most studied member of the ω-HXTX-1 family of insecticidal toxins isolated from the venom of the Australian funnel web spider *Hadronyche versuta* [9]. It has been shown to be highly toxic by injection to a wide range of insects [1]–[3], [9]. Toxins of the ω-HXTX-1 family contain three conserved disulphide bonds that form an inhibitor cystine knot motif that is critical for toxin activity. We therefore used *Pichia pastoris*, a host capable of correctly forming disulphide cross-links, to create a fusion protein containing Hv1a linked to the N-terminal region of the lectin GNA. The use of a secretory signal enabled facile purification of recombinant fusion protein from fermented culture supernatants. As observed previously for SFI1/GNA and ButaIT/GNA [13], [14], some cleavage of the Hv1a/GNA fusion protein occurred during expression, despite the use of a protease-deficient host strain. Differences were observed in the degree of proteolysis and cleavage patterns (as assessed by Western blot analysis) for the three fusion proteins although all appear to be prone to cleavage between the N-terminal region of the toxin sequence and the C-terminus of GNA. For Hv1a/GNA, a single cleavage site is indicated by the presence of two major proteins in purified fractions, corresponding to intact fusion protein and GNA protein from which the Hv1a toxin has been cleaved. Nevertheless, the majority of expressed Hv1a/GNA was present as intact fusion protein, as evidenced by molecular mass on SDS-PAGE gels and positive immunoreactivity with anti-GNA and anti-Hv1a antibodies. The C-terminus of Hv1a (residues 33–36) includes the sequence VKRC, which is similar to the signal sequence (EKRE) present in the α-factor signal sequence expression vector that is cleaved between R and E by the KEX2 gene product. Further analysis is required to establish whether this or another site is the precise location of cleavage between the Hv1a peptide and GNA protein.

Previously reported values for toxicity by injection of recombinant and synthetic Hv1a are highly variable, even when considering different species of the same genus. For example, the ED_50_ reported for synthetic Hv1a against the cotton bollworm *Heliothis armigera* is 3 nmol/g [7], which is more than 10-fold higher than the PD_50_ dose of 250 pmol/g reported for the tobacco hornworm *Heliothis virescens* [8]. In our hands, the doses of injected recombinant Hv1a and Hv1a/GNA required to induce flaccid paralysis and significant mortality of fifth stadium *M. brassicae* larvae were comparable (50–100 µg toxin/g insect equivalent to 12–25 nmoles/g), suggesting that Hv1a activity is not significantly compromised by C-terminal linkage to GNA. However, these doses are somewhat higher than those typically reported for recombinant Hv1a (e.g., LD_50_ of 77 pmol/g and 716 pmol/g respectively for the housefly *Musca domestica* and lone star tick *Amblyomma americanum*; [10]). Differences in the toxicity of Hv1a towards different species must, to a large degree, be determined by differences in the ability of the toxin to disrupt ion channel function. However, variability also derives from the use of different toxicity parameters (e.g., LD_50_, ED_50_ and PD_50_), different sources of toxin (i.e. synthetic, recombinant or native peptide) and the suitability and/or ease of injection. In previous studies we assessed the injection toxicity of the spider toxin SFI1 and the scorpion toxin ButaIT, when fused to GNA, towards larvae of the tomato moth *Lacanobia oleracea* and the cotton leafworm *Spodoptera littoralis*. Whilst SFI1/GNA was more toxic than ButaIT/GNA towards *L. oleracea* [12], [14], ButaIT/GNA was more toxic than SFI1/GNA towards *S. littoralis* [16]. In addition, ButaIT/GNA was found to be more generally toxic than SFI1/GNA when tested against a range of insect pests including lepidopteran larvae, dipteran adults, coleopteran adults and larvae and dictyopteran nymphs [16]. Variability in the susceptibility of different insect species prevents strict comparative analysis. However, we note that the amounts of Hv1a/GNA necessary to cause toxicity to *M. brassicae* larvae (50–100 µg toxin/g insect causing >50% mortality) are comparable to the amounts required for other GNA-toxin fusion proteins (i.e., SFI1/GNA: 20–125 µg toxin/g insect causing >50% mortality in *Lacanobia oleracea* [12]; ButaIT/GNA: 50–135 µg toxin/g insect causing >50% mortality in *Spodoptera littoralis* [16]).

### Fusion to GNA Massively Enhances the Oral Toxicity of Hv1a

Hv1a has been reported to be orally active against ticks, and it appears to be orally active against the lepidopteran *Spodoptera littoralis* and *Helicoverpa armigera* when expressed in plants [10], [17]. However, we found that Hv1a alone was not orally active when fed to fifth stadium *M. brassicae* larvae. This is consistent with the observation that the LD_50_ for Hv1a in the sheep blowfly *Lucilia cuprina* is 90-fold lower when the toxin is delivered *per os* compared with injection (V. Herzig and G.F.K, unpublished data). In striking contrast, the Hv1a/GNA fusion protein was orally toxic towards *M. brassicae* larvae in both cabbage leaf disc and droplet feeding assays. High levels of mortality and reduced growth were observed for second instar larvae exposed to discs coated with purified fusion protein. The oral toxicity observed in these assays must be a result of the Hv1a/GNA fusion protein, since GNA at a comparable dose did not reduce survival (although a reduced effect on larval growth was observed). These results are comparable to previously published data for the oral insecticidal activity of SFI1/GNA, and growth inhibition of GNA, towards *L. oleracea* larvae [12].

The consumption of droplets containing 40 µg of Hv1a/GNA fusion protein by fifth stadium larvae was seen to result in a complete cessation of feeding and larvae appeared relatively immobile, consistent with the previously described paralytic activity of the toxin [1], [9]. Larvae failed to survive to pupation following droplet consumption of a total of 160 µg of fusion protein over four days. By contrast, larvae exposed to droplets containing an equivalent dose of Hv1a showed no evidence of reduced feeding or paralysis and all survived to pupation. The absence of oral toxicity for Hv1a contrasts with the previous results reporting 100% mortality of *Heliothis armigera* and *S. littoralis* exposed to transgenic tobacco expressing Hv1a [17]. One possibility is that natural insecticidal compounds produced by these plants might produce disturbances in the insect gut epithelium and thereby act synergistically with Hv1a to improve its oral activity. Khan and co-workers [17] also reported contact insecticidal activity for Hv1a, although in their assays the fusion protein was applied topically in a solution containing high levels of imidazole, a compound known to have contact insecticidal activity [18].

### GNA Mediates Delivery of Hv1a to Insect CNS

Most spider toxins act peripherally at neuromuscular junctions but Hv1a acts at sites within the central nervous system [1], [8]. Bloomquist, 2003 previously demonstrated that Hv1a is able to cross the nerve sheath; whereas Hv1a acted instantaneously in *Drosophila melanogaster* nerve preparations that had been transected to facilitate toxin penetration, an 18 minute delay in the blockage of nerve firing occurred when intact nerve preparations were used and this delay was consistent with the time taken to observe paralysis following injection of the toxin. Surprisingly, Western blot analysis of nerve chords dissected from insects injected with GNA and Hv1a/GNA indicated that GNA binds to the central nerve chord of lepidopteran larvae and is therefore capable of mediating the delivery of Hv1a to sites of action within the CNS.

Further direct evidence for GNA localization to CNS was provided by fluorescence imagery of nerve chords dissected from larvae that had been injected with, or fed on, FITC-labelled proteins. That GNA binds to mannose-containing membrane-bound polypeptides was indicated by intense fluorescence of the nerve chord sheath and also by reduced binding in tissues extracted from insects injected with GNA that had been pre-incubated with mannose.

Neurophysiological studies with cockroaches, lepidopteran and dipteran larvae have indicated that Hv1a impairs ganglionic neural transmission, rather than conductance along the nerve chord. The characteristic delay in paralysis observed after injection of the toxin is thought to be attributable to the time required for the toxin to cross the nerve sheath and enter the CNS [1], [8]. The results presented here suggest that GNA may help to localise covalently attached insecticidal neurotoxins, such as Hv1a, to the CNS of exposed insects and thereby facilitate toxin action within the CNS.

In conclusion, the data presented here indicates that GNA not only mediates delivery of insecticidal peptides across the insect gut but that it is also capable of delivering peptides to the insect central nervous system. In the case of Hv1a, the massive improvement in oral activity upon fusion to GNA can be attributed to both of these properties. Many insecticidal peptides have been isolated from arachnid venoms [9], [19], [20], and fusion to GNA would appear to provide a general mechanism for dramatically enhancing their oral activity. GNA-toxin fusion proteins could be used for crop protection either as exogenously applied treatments or as endogenous proteins expressed in transgenic plants or entomopathogens.

## Materials and Methods

### Materials and Recombinant Techniques

General molecular biology protocols were as described in [21] except where otherwise noted. Subcloning was carried out using the TOPO cloning kit (pCR2.1 TOPO vector; Invitrogen). *Pichia pastoris* SMD1168H (protease A deficient) strain, the expression vector pGAPZαB, and Easycomp *Pichia* transformation kit were from Invitrogen. Oligonucleotide primers were synthesised by Sigma-Genosys Ltd. T4 polynucleotide kinase was from Fermentas. Restriction endonucleases, T4 DNA ligase, and *Pfu* DNA polymerase were supplied by Promega. Plasmid DNA was prepared using Promega Wizard miniprepkits. GNA was produced as a recombinant protein in yeast using a clone generated as previously described [22]. Anti-GNA antibodies (raised in rabbits), were prepared by Genosys Biotechnologies, Cambridge, UK. Anti-Hv1a polyclonal antibodies (raised in rabbits) were prepared by the Institute of Medical and Veterinary Science, Adelaide, Australia. Recombinant Hv1a was prepared as described previously [2], [4].

All DNA sequencing was carried out using dideoxynucleotide chain termination protocols on Applied Biosystems automated DNA sequencers by the DNA Sequencing Service, School of Biological and Biomedical Sciences, University of Durham, UK. Sequences were checked and assembled using Sequencher software running on Mac OS computers. The Hv1a/GNA sequence has been deposited in Genbank (#1527166).

#### Assembly of expression constructs for production of Hv1a/GNA fusion protein

The Hv1a amino acid sequence (UniProtKB P56207) was used as the basis for assembly of a synthetic Hv1a gene. Codon usage was optimised for expression in yeast (www.yeastgenome.org/community/codonusage.shtml). The coding strand was subdivided into two fragments and the complementary strand was subdivided into three fragments, such that the coding fragments overlapped the complementary strand fragments by 21 bases. Five oligonucleotides based on these fragments were synthesised and used to assemble the mature Hv1a coding sequence (Table 1). All primers were individually 5′-phosphorylated using T4 polynucleotide kinase. An equimolar solution of 100 pmol of each phosphorylated primer was boiled for 10 min to denature secondary structures, then the solution was slowly cooled to room temperature (RT) to allow the primers to anneal. After addition of T4 DNA ligase, annealed oligonucleotides (in ligase buffer) were left to anneal for 15 h at 4°C. To obtain sufficient DNA for cloning into the yeast expression vector pGAPZαB, the Hv1a coding sequence was amplified by PCR using primers containing 5′ *Pst*I and 3′ *Not*I restriction sites. Following amplification, gel purification and restriction digest, the PCR product was ligated into a previously generated yeast expression construct [14] containing the mature GNA coding sequence (amino acids 1–105 derived from LECGNA2 cDNA; [23]) to create the plasmid Hv1a/GNA-pGAPZαB. The sequence of the Hv1a/GNA expression construct has been given the accession number JQ898015 by GenBank.

### Expression and Purification of Hv1a/GNA Fusion Protein

Plasmid Hv1a/GNA-pGAPZαB DNA was transformed into chemically competent *P. pastoris* cells (strain SMD1168H) according to protocols supplied by Invitrogen. Transformants were selected by plating on medium containing zeocin (100 µg/ml). A clone expressing recombinant Hv1a/GNA was selected for production by bench-top fermentation by Western analysis using anti-GNA (1∶3300 dilution) antibodies of supernatants from small-scale cultures grown at 30°C for 2–3 days in YPG medium (1% w/v yeast extract; 2% w/v peptone; 4% v/v glycerol; 100 µg/ml zeocin) (results not shown).

For protein production, *P. pastoris* cells expressing Hv1a/GNA fusion protein or GNA encoding sequences were grown in a BioFlo 110 laboratory fermenter. Briefly, 3×100 ml YPG cultures (grown for 2–3 days at 30°C with shaking) were used to inoculate 3 l of sterile minimal media supplemented with PTM1 trace salts [24], [25] Cultivation was conducted at 30°C, pH 4.5–5.0, 30% dissolved oxygen (cascaded agitation 250–750 rpm) with a glycerol feed (5–10 ml/h; 1.3 l over 72 h). Secreted proteins were separated from cells by centrifugation (30 min at 7500 *g*, 4°C). NaCl was added to the supernatant to a final concentration of 2 M. Recombinant proteins were purified by hydrophobic interaction chromatography on a phenyl-Sepharose (Amersham Pharmacia Biotech) column (1 cm dia., 25 ml), run at 2 ml/min. After loading, the phenyl-Sepharose column was washed with 2 M NaCl and a linear salt gradient (2 M–0 M NaCl) applied over 60 min. Recombinant Hv1a/GNA eluted at ∼1 M NaCl. Fractions containing purified proteins (analysed by SDS-PAGE) were then pooled, dialysed against distilled water and lyophilised. Lyophilised fusion protein and GNA were subject to gel filtration on Sephacryl S-200 columns (1.6 cm diameter, 90 cm length, flow rate 0.3 ml/min) to remove high molecular weight yeast proteins as described previously [14]. Fractions containing purified recombinant proteins were again dialysed and lyophilised, or desalted and concentrated using Microsep TM centrifugal concentrators (VivaScience AG, Hannover, Germany).

### Electrophoresis and Western Blotting

Proteins were routinely analysed by SDS-PAGE (17.5% acrylamide gels). Samples were prepared by adding 5× SDS sample buffer (containing 10% β-mercaptoethanol) and boiling for 10 min prior to loading. Gels were either stained with Coomassie blue or transferred to nitrocellulose for Western blotting using a Biorad Trans-blot SD semi dry transfer cell according to the manufacturer’s recommendations. Western blotting of recombinant proteins and larval samples (haemolymph and nerve chord) using anti-GNA (1∶3300 dilution) or anti-Hv1a (1∶1000 dilution) antibodies was carried out as described [26].

### FITC Labelling

Recombinant GNA, Hv1a/GNA, and ovalbumin (control) were fluorescently labelled with a 2∶1 molar excess of fluorescein isothiocyanate (FITC, Sigma). Recombinant proteins (1 ml) were re-suspended at 2 mg/ml in 500 mM carbonate buffer pH 9.0 then incubated with 50 µl FITC (1 mg/ml in DMSO) with rotation for 4 h at RT, under dark conditions. Samples were dialysed against phosphate-buffered saline (PBS pH 7.4) at RT to remove excess FITC. FITC labelling of Hv1a was unsuccessful, presumably due to the scarcity of primary amines available for FITC attachment.

### Insect Rearing

*M. brassicae* were originally obtained from cultures held at the Food and Environment Research Agency (FERA) and were reared at the University of Durham continuously on artificial diet [27] at 22–25°C under a 16 h∶8 h light:dark regime.

### Injection Bioassays

Purified recombinant Hv1a peptide and Hv1a/GNA were tested for biological activity by injecting 4–5 µl of aqueous samples (lyophilised protein re-suspended in PBS) into newly eclosed fifth stadium *M. brassicae* larvae (40–70 mg). For each concentration tested, 10–20 larvae were injected and toxic effects were monitored over 4 days. PBS was injected as a negative control. Recombinant GNA is known to have no effect upon *M. brassicae* larvae when injected at up to 200 µg/larva (unpublished data).

### Feeding Bioassays

#### Droplet feeding assays: *M. brassicae*

Several droplet-feeding assays were conducted to assess the oral activity of Hv1a/GNA towards M. brassicae fourth and fifth stadium larvae. Final sample numbers were relatively small (n = 7–8 per treatment) as larvae were reluctant to ingest daily droplets and insects that did not consume a full 5-µl droplet were discarded from data sets. Two representative assays are described herein.

#### Droplet assay 1

Newly moulted fifth stadium larvae were fed daily for 4 days with a 5-µl droplet containing 40 µg of Hv1a/GNA or 9.6 µg of Hv1a toxin in 1×PBS and 10% sucrose solution. Control larvae were fed on droplets containing 40 µg bovine serum albumin (BSA). To encourage droplet consumption, larvae were starved for ∼2–3 h prior to feeding. Larval weight was recorded daily ∼1 h after droplet feeding. Treated larvae were placed individually in ventilated plastic pots (250 ml) with standard artificial diet. After 4 days of daily droplet feeding, larvae were maintained on optimal diet until the onset of pupation.

#### Droplet assay 2

Newly moulted fifth stadium larvae were fed on a single 5-µl droplet containing 40 µg of Hv1a/GNA or 40 µg BSA (control) in 1×PBS and 10% sucrose. Larvae were maintained as described above and weights recorded daily for 10 days.

#### Leaf disc assays: *M. brassicae*

The oral activity of Hv1a/GNA was further tested by feeding second instar M. brassicae larvae on cabbage (Brassicae oleracea) discs coated with purified fusion protein at concentrations of 0.2% w/w and 0.1% w/w (i.e., 10 mg/5 g and 5 mg/5 g leaf wet weight, respectively) or recombinant GNA at 0.2% w/w. Discs (∼20 mm dia., 140 mg fresh wt.) were prepared by adding droplets of protein (re-suspended in 0.5×PBS and 0.1% v/v Tween) onto upper and lower surfaces of discs and air dried. Control discs were prepared with 0.5× PBS, 0.1% v/v Tween. Larvae were reared from hatch for 72 h on non-treated cabbage and then placed into ventilated plastic pots (250 ml) containing coated leaf discs and moist filter paper to prevent dessication. Freshly prepared discs were provided every 2–3 days. Two replicates of 10 larvae per treatment were assayed. Survival was recorded for 10 days. To minimise handling time, larval weights were recorded on days 4, 7, and 10.

### Haemolymph Extraction and Nerve Chord Dissection

Haemolymph samples were extracted and prepared for Western analysis [12] from day 2 fifth instar larvae fed for 24 h on diet containing Hv1a/GNA at 2 mg/5 g wet wt. (∼2% dietary protein). Typically, aliquots of two replicate samples containing pooled haemolymph (3–5 larva per sample) were run on SDS-PAGE gels and analysed by immunoblotting using anti-GNA antibodies. To investigate if GNA or Hv1a/GNA were localized to the CNS after oral delivery or injection, nerve chords were analysed by one of two methods. Nerve chords were dissected from sixth stadium larvae 4–24 h after injection or after being fed on droplets containing 20–50 µg GNA or fusion protein. Nerve tissue was subsequently analysed by Western blotting or visualised by fluorescent microscopy (section 2.11). Nerve chords were dissected as follows. Pre-chilled larvae were immersed in ice-cold distilled water prior to making a ventral incision from the tail to the head capsule. The resulting flaps of cuticle were fixed with pins into dissecting wax. The entire gut was carefully removed and the head capsule split to expose the terminal brain ganglia. Intact nerve chord and brain was then separated (using scissors) from the cuticle and head capsule and immersed immediately either in SDS sample buffer for Western analysis or in 3.7% w/v paraformaldehyde (PFA) for microscopy.

### Fluorescent Microscopy

Nerve chords were extracted from sixth stadium larvae 4 h after injection of ∼10 µg of FITC-labelled GNA or FITC-labelled Hv1a/GNA. Larvae were also injected with GNA that had been pre-incubated for 1 h at RT with 0.2 M mannose (methyl α-D-mannopyranoside). Nerve chords were also extracted from larvae after feeding on artificial diet containing FITC-labelled GNA or FITC-labelled Hv1a/GNA such that each larva consumed 50–100 µg labeled protein. Control treatments included FITC-labelled ovalbumin (10 µg per injection, 50–100 µg by ingestion) and FITC alone (0.5 µg per injection, 2.5 µg by ingestion). Following dissection and immersion in PFA (30–60 min), nerve chords were washed 3×in ice cold PBS (15 min per wash), mounted onto glass slides and overlaid with coverslips. Nerve chords were visualized using a fluorescent microscope (Nikon) under FITC filter (absorbance 494 nm; emission 521 nm) and images were captured in OpenLab.

### Statistical Analysis

Data were analysed using Prism 5.0 (GraphPad Software Inc.). Kaplan–Meier insect survival curves were compared using Mantel–Cox log-rank tests. Insect weights were compared using either Student’s *t*-tests or one-way analysis of variance (ANOVA), followed by Tukey–Kramer *post hoc* means separation. The accepted level of significance was *P<*0.05 in all cases.
